# Mean-return-time phase of a stochastic oscillator provides an approximate renewal description for the associated point process

**DOI:** 10.1007/s00422-022-00920-1

**Published:** 2022-02-15

**Authors:** Konstantin Holzhausen, Lukas Ramlow, Shusen Pu, Peter J. Thomas, Benjamin Lindner

**Affiliations:** 1 Bernstein Center for Computational Neuroscience Berlin, Philippstr. 13, Haus 2, 10115 Berlin, Germany; 2 Physics Department of Humboldt University Berlin, Newtonstr. 15, 12489 Berlin, Germany; 3 Department of Biomedical Engineering, 5814 Stevenson Center, Vanderbilt University, Nashville, TN 37215 USA; 4 Department of Mathematics, Applied Mathematics, and Statistics, 212 Yost Hall, Case Western Reserve University, 10900 Euclid Avenue, Cleveland, Ohio USA

**Keywords:** Stochastic neuron models, Phase description, Stochastic oscillations, Interspike interval statistics

## Abstract

Stochastic oscillations can be characterized by a corresponding point process; this is a common practice in computational neuroscience, where oscillations of the membrane voltage under the influence of noise are often analyzed in terms of the interspike interval statistics, specifically the distribution and correlation of intervals between subsequent threshold-crossing times. More generally, crossing times and the corresponding interval sequences can be introduced for different kinds of stochastic oscillators that have been used to model variability of rhythmic activity in biological systems. In this paper we show that if we use the so-called mean-return-time (MRT) phase isochrons (introduced by Schwabedal and Pikovsky) to count the cycles of a stochastic oscillator with Markovian dynamics, the interphase interval sequence does not show any linear correlations, i.e., the corresponding sequence of passage times forms approximately a renewal point process. We first outline the general mathematical argument for this finding and illustrate it numerically for three models of increasing complexity: (i) the isotropic Guckenheimer–Schwabedal–Pikovsky oscillator that displays positive interspike interval (ISI) correlations if rotations are counted by passing the spoke of a wheel; (ii) the adaptive leaky integrate-and-fire model with white Gaussian noise that shows negative interspike interval correlations when spikes are counted in the usual way by the passage of a voltage threshold; (iii) a Hodgkin–Huxley model with channel noise (in the diffusion approximation represented by Gaussian noise) that exhibits weak but statistically significant interspike interval correlations, again for spikes counted when passing a voltage threshold. For all these models, linear correlations between intervals vanish when we count rotations by the passage of an MRT isochron. We finally discuss that the removal of interval correlations does not change the long-term variability and its effect on information transmission, especially in the neural context.

## Introduction

A number of biological systems of rather different nature display stochastic oscillations. The calcium concentration within cells (Skupin et al. 2008), the deflection of mechanical organelles like the hair bundle (Martin et al. 2003), the position of molecular motors (Plaçais et al. 2009), the membrane potentials of neurons (Bryant et al. 1973; Walter et al. 2006), and even the number of individuals in biological populations (McKane and Newman 2005) can all show a quasi- rhythmic behavior that is shaped and in some cases even only enabled by randomness. Stochastic models for such kind of oscillations are diverse as well, including harmonic oscillators with damping and fluctuations (Uhlenbeck and Ornstein 1930; Schimansky-Geier and Zülicke 1990), randomly perturbed limit-cycle systems (see, Ebeling et al. 1986 for an early example), and noisy excitable (Lindner et al. 2004) or heteroclinic systems (e.g., Giner-Baldo et al. 2017).

Most models are complicated (multidimensional, nonlinear, and stochastic) and even the calculation of such fundamental statistics as the stationary probability density or the mean rotation period is difficult. Hence, researchers have attempted reduced descriptions that would capture the salient features of the system and enable, for instance, the analysis of coupled oscillator systems. In the deterministic case (without noise) the most successful simplification is a phase description: to every point in the multidimensional phase space we assign a phase, reducing in this way a multidimensional system to a one-dimensional description. The great success of this mapping is that weak interactions between nonlinear oscillators can be efficiently described in terms of the phase response curve (Hoppensteadt and Izhikevich 1997).

To generalize the concept of a phase to the stochastic case is nontrivial, and different notions of phase have been suggested. The mean-return-time (MRT) phase by Schwabedal and Pikovsky (2013) is a generalization of the stroboscopic definition of a deterministic phase; while the asymptotic phase introduced by Thomas and Lindner (2014) is a generalization of the long-term properties of two phase points in the deterministic case. Here we focus on the first definition of phase, the MRT phase: Points in the phase space belong to the same phase (they are on the same isochron) if the mean time to return to the same curve after one rotation is equal to the mean period of the oscillator. To implement this condition according to this algorithmic definition is not as straightforward as it may sound. More recently, Cao et al. (2020) proposed an analytical definition for a special class of planar white-noise-driven oscillators, which is based on the well-known partial differential equation for the mean-first-passage time with an unusual jump condition.

Another simplifying approach to oscillatory systems is to associate a point process with the repetitive features of the system: In neurons, for instance, upcrossings of a voltage threshold have been used to define a spike train or, equivalently, an ordered sequence of interspike intervals (ISIs); in heart dynamics, the intervals between heartbeats have been analyzed in a similar way. Besides the statistical distribution of the single intervals, its mean, variance, coefficient of variation, skewness, etc., correlations among the intervals have attracted attention because they may betray interesting dynamical features of the system or the driving stimuli. Most often, one focusses on the linear correlations as quantified by the serial correlation coefficient (SCC)

1 $$\begin{aligned} \rho _k=\frac{\left\langle (I_i-\left\langle I_i \right\rangle ) (I_{i+k}-\left\langle I_{i+k} \right\rangle ) \right\rangle }{\left\langle (I_i-\left\langle I_i \right\rangle )^2 \right\rangle }, \end{aligned}$$

where the average can be taken over the sequence of intervals (i.e., over the index *i*) or, equivalently, over an ensemble of spike trains (then *i* would be fixed). For a stationary sequence of intervals, the SCC compares the covariance between two intervals lagged by an integer *k* to the variance of the single interval (this yields a number between -1 and 1). If intervals are independent, as is the defining property of a *renewal point process*, $\rho _k=0$ for $k>0$. (We always have $\rho _0=1$ by definition.) Note that this conclusion cannot be reversed: A point process with vanishing SCC can still display nonlinear correlations and there might be a statistical dependence among its intervals. Hence, strictly speaking, a process with $\rho _k=0,\; \forall k>0$ is *not necessarily* a renewal process. Still, because $\rho _k$ is the almost exclusively used measure of nonrenewal behavior, we may still regard a spike train with vanishing linear correlations as being *approximately renewal*.

In neurons, nonrenewal behavior, i.e., nonvanishing ISI correlations may emerge because of slow (Lindner 2004; Schwalger and Schimansky-Geier 2008) or quasi-rhythmic (Bauermeister et al. 2013) stochastic stimuli, in networks due to refractoriness of presynaptic neurons, short-term synaptic depression (Schwalger et al. 2015), and, last but not least, spike-frequency adaptation (Liu and Wang 2001; Chacron et al. 2001); see Farkhooi et al. (2009); Avila-Akerberg and Chacron (2011) for reviews on experimental data of the SCC and its implications for signal transmission. Interbeat intervals in heart dynamics show correlations as well due to the often highly nonlinear and complex dynamics, see, e.g., Kim et al. (2019); Goldberger et al. (2002).

We note that the most studied stochastic model of spike generation, the one-dimensional integrate-and-fire model driven by white noise would generate a renewal process—the reset of the voltage after reaching a threshold would eliminate any memory of past intervals and the driving noise is uncorrelated by assumption and cannot carry any memory either. In contrast, multidimensional stochastic neuron models (which include, for instance, dynamical variables for spike-frequency adaptation and/or colored noise) can generate richer (nonrenewal) spike statistics.

In this paper, we report the remarkable observation that counting rotations in terms of the MRT phase in planar white-noise-driven oscillators leads to a sequence of interphase intervals (IPIs), for which linear correlations vanish. Put differently, if we count spikes not with a standard threshold but rather by the passing of an MRT isochron, the associated point process will be (at least approximately) a renewal process.

In the next section, we give the general rationale for this result. We then look at specific examples in Sect. 3. In Sect. 3.1 we analyze an isotropic noise-driven oscillator with two stable limit cycles that, counted with a conventional threshold, generates an ISI sequence with pronounced positive correlations. In Sect. 3.2 we test our idea for an integrate-and-fire model with spike-frequency adaptation that is well known for its negative ISI correlations (Liu and Wang 2001). Finally, in Sect. 3.3 we look at a conductance-based neuron model with channel noise that has weak positive ISI correlations if spikes are generated by upcrossings of a voltage threshold. In all cases, counting rotations as passings of the MRT isochron leads to an IPI sequence with vanishing correlation coefficients. We conclude the paper with a brief discussion of the implications of our result for modeling stochastic oscillations.

## Model class and general result

Here we introduce the general model of a stochastic oscillator with white noise and recapitulate how phase lines and corresponding crossing times forming a point process can be defined. We discuss the salient feature of the mean-return-time phase and argue why linear correlations among the corresponding interphase intervals should vanish.

### The general oscillator model

We consider an *n*-dimensional nonlinear stochastic system, given in terms of a system of Langevin equations:

2 $$\begin{aligned} \dot{\mathbf {x}}=f(\mathbf {x})+g(\mathbf {x})\varvec{\xi }(t). \end{aligned}$$

Here $f(\mathbf {x})$ is the *n*-dimensional drift vector, $g(\mathbf {x})$ is an $n\times k$ matrix (where *k* can be larger than *n*), and $\varvec{\xi }(t)$ a *k*-dimensional vector of white Gaussian noise processes with vanishing mean values and correlation functions

$$\begin{aligned} \left\langle \xi _i(t)\xi _j(t') \right\rangle =\delta _{ij} \delta (t-t'),\;\; i,j=1,\dots k\ge n. \end{aligned}$$

Here $f(\mathbf {x})$ is the *n*-dimensional drift vector, $g(\mathbf {x})$ is an $n\times k$ matrix. For technical reasons, we require that the matrix $g(\mathbf {x})g(\mathbf {x})^\intercal$ be invertible everywhere (see Cao et al. (2020)). If the noise is multiplicative ($g(\mathbf {x})\ne$ const), it is always interpreted in the sense of Itô. Furthermore, for certain types of models (integrate-and-fire neurons), an additional reset rule for the trajectory applies if it reaches certain boundaries. Much of what we discuss here is illustrated in terms of two-dimensional (planar) systems with $n=2$ but can be generalized to higher dimensions. At the risk of stating the obvious: the above system of Langevin equations describes a Markov process $\mathbf {x}(t)$ (irrespective of whether a reset rule applies or not).

**Fig. 1:**
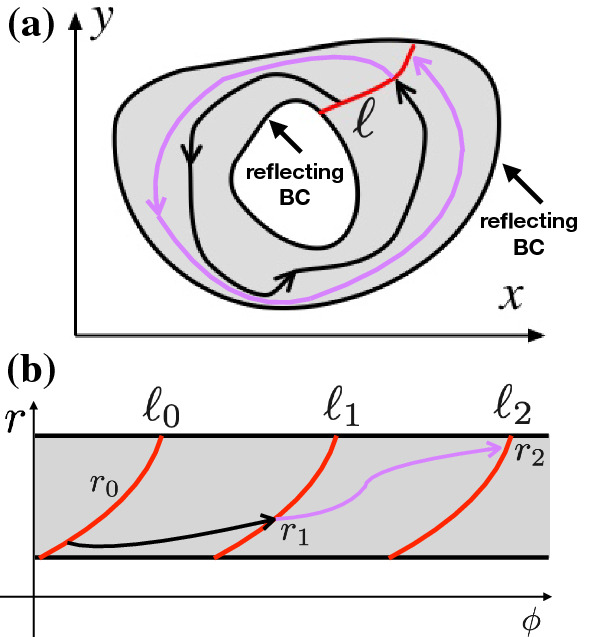
Mapping from the stochastic oscillator dynamics in Cartesian coordinates (**a**) to effective angle-radius coordinates (**b**). For simplicity we consider a dynamics that is constrained to a ring-like domain by imposed reflecting boundaries on an inner and an outer circle (or topologically equivalent lines as in the sketch). Simple connections between these boundaries define a phase line $\ell$. The return-time problem in Cartesian variables poses some subtle problems that can be removed by considering the passage-time problem between copies of the phase line in angle-radius coordinates ($\ell _0, \ell _1, \ell _2$ in (**b**). We show two rotations (first in black, second in purple) in (**a**) and (**b**). Technically, the mapping from (**a**) to (**b**) may involve as an intermediate step the mapping to a true annulus and from the true annulus to polar coordinates in (**b**) (see Cao et al. (2020))

We assume that the system undergoes stochastic oscillations, i.e., it performs randomly timed rotations around a center core and remains within an annulus-like domain (cf. Fig. 1); for integrate-and-fire models this is a bit more complicated because the trajectory remains within a certain cutout of the annulus—the reset rule shortcuts a part of it but for the moment we leave this complication aside. In the general case, as a helpful construction, we impose reflecting inner and outer boundaries. Both boundaries of the domain are chosen such that reflections are rare events and the main share of probability lies far from the boundaries. In many cases we may also perform the limit in which the inner and the outer boundary shrink to zero or go to infinity, respectively (see Holzhausen et al. (2022); Holzhausen (2021) for some examples). Here we are not interested in the mathematical generality of the result but assume that the considered system is sufficiently non-pathological such that the exact values of the boundaries are not important (except for the behavior close to those boundaries). We furthermore assume that for the stochastic oscillator, the sets of constant phase (such as the MRT phase or the asymptotic phase) are given by simple manifolds that can be parametrized by polar coordinates as $(r,\phi (r))$; this has been the case for all examples studied by us and co-authors in the past (Thomas and Lindner 2014, 2015, 2019; Cao et al. 2020; Pérez-Cervera et al. 2021).

For a planar system we can define a simple connecting curve $\ell$ (or a connecting $(n{-}1)$-dimensional manifold for an *n*-dimensional system) between the inner and the outer boundaries. We count rotations by the crossings of $\ell$, or, put differently, the *return* to this curve *after the completion of a rotation*. The latter condition is important: Crossings of a curve are a subtle issue in dynamical systems driven by white noise because even if we restrict the crossings to be counted only when occurring into the direction of rotation, there will be infinitely many of them in a finite time if we count them in a naive way without the condition of the completed rotation (for the general problem of the number of crossings for a stochastic process, see Stratonovich (1967)).

Helpful in this respect is a mapping of the trajectory in Cartesian coordinates to a first-passage-time problem in a transformed space with an angle variable and a number of radius variables. This is evident in the two-dimensional case, in which we just transform to effective angle-radius variables, as illustrated in Fig. 1. The connecting curve $\ell$ in Cartesian coordinates can now be numbered, according to the number of completed rotations, e.g., $\ell _0(r,\phi ),\ell _1(r,\phi ),\dots$. The return of the trajectory starting at $\ell$ to the very same curve $\ell$ in Cartesian coordinates is mapped in polar coordinates to a passage from the curve $\ell _0=(\phi (r),r)$ to a copy of the curve at $\ell _1=(\phi (r)+2\pi ,r)$.

For certain obvious choices of the curve (spoke of a wheel or, in the neural models, a voltage threshold), we call the crossing times of the curves $\ell _i$ the *spike times* $t_i$ and the intervals between adjacent spike times the *interspike intervals* $I_i=t_i-t_{i-1}$. These intervals

3 $$\begin{aligned} ..., I_{i-1}, I_i, I_{i+1}, ... \end{aligned}$$

form an ordered sequence of stochastic variables that will in general be correlated, i.e., the correlation coefficient, defined in (1), displays nonvanishing values, $\rho _k\ne 0$ for $k>0$. We expect that correlations depend on the lag between two intervals and that these correlations vanish as the lag goes to infinity ($\lim \limits _{k\rightarrow \infty }\rho _k =0$).

The stationary mean value of the intervals $I_i$

4 $$\begin{aligned} {\bar{T}}=\lim _{N\rightarrow \infty } \frac{1}{N}\sum _{i=1}^N I_{i}, \end{aligned}$$

the mean rotation period, is independent of the specific choice of $\ell$. To see this, consider the relation of ${\bar{T}}$ to the winding number, i.e., the mean number of rotations per time unit $\nu =\left\langle N(T) \right\rangle /T$ obtained by time averaging over a long window (0, *T*):

$$\begin{aligned} \nu =\lim _{T\rightarrow \infty } \frac{N}{T}=\lim _{N\rightarrow \infty } \left( t_0+\frac{1}{N}\sum _{i=1}^{N-1} I_{i}+t_N\right) ^{-1}=\frac{1}{{\bar{T}}}, \end{aligned}$$

Here, we have used that what we find in the denominator is essentially the definition of the mean rotation period. (The effect of the start and final intervals $t_0$ and $t_N$ becomes negligible for $N\rightarrow \infty$.) Because the winding number $\nu$ cannot depend on the specific way we count the rotation, also ${\bar{T}}$ (its inverse) cannot depend on the shape of $\ell$ (as long as it faithfully counts every single rotation at some point).

### The MRT phase and the associated point process

The MRT phase is defined as a set of special phase lines $\ell _{\text {MRT},k}$, such that for all points which start with a given phase, i.e., on the isochron $\ell _{\text {MRT},0}=(r,\phi _\text {MRT}(r))$, the mean time to reach the very same isochron again after one rotation (or in polar coordinates the copy $\ell _{\text {MRT},1}=(r,\phi _\text {MRT}(r)+2\pi )$ is equal to the mean rotation time of the oscillator, irrespective of the starting point on the isochron, $(r,\phi _\text {MRT}(r))$,

5 $$\begin{aligned} \left\langle T(\phi _\text {MRT}(r) \rightarrow 2\pi +\phi _\text {MRT}(r)) \right\rangle ={\overline{T}} \; \forall \; r_-<r<r_+ \end{aligned}$$

Note that the target radius variable upon return to $\ell$ can have an arbitrary value; the defining property of this special isochron is that there is no dependence of the average interval on the radius of the starting point on $\ell _0$. For planar oscillators, Cao et al. (2020) showed that this definition uniquely determines the phase mapping (apart from a trivial off-set of the phase, of course). We also note that in the limit of vanishing noise, this corresponds to the classical phase of the oscillator (if it exists).^1^

Subsequent passing of the isochrons $\ell _i$ at times $t_i$ can be used to define a sequence of special interspike intervals that we call in the following the *interphase intervals* (IPIs) $T_i=t_i-t_{i-1}$:

6 $$\begin{aligned} \dots , T_{i-1},T_i,T_{i+1}\dots . \end{aligned}$$

On the notation: we reserve the letter $I_i$ for a general interval (including the interspike interval), while $T_i$ is specifically the interval for the MRT-phase-line crossings, i.e., the IPI.

The main result of our paper is that for the sequence in (6), there are no linear correlations, i.e., $\rho _k=0$ for $k>0$. Why should this be the case?

### Why we can expect that IPI correlations vanish

We focus now on the passages from an arbitrary phase line $\ell _0$ (not necessarily the MRT phase) to its $2\pi -$shifted copy $\ell _1$ and from $\ell _1$ to $\ell _2$, i.e., on the two subsequent passage times $I_1$ and $I_2$. The covariance between these two intervals $\left\langle I_1 I_2 \right\rangle -\left\langle I_1 \right\rangle \left\langle I_2 \right\rangle$ is the central piece of the correlation coefficient $\rho _1$. We can write the stationary average of the product of the intervals as follows

7 $$\begin{aligned} \left\langle I_1 I_2 \right\rangle= & {} \int dr_0 \int dr_1 \int dr_2 \int dI_1 \int dI_2\nonumber \\&\times I_1 I_2 P(I_1,I_2,r_0, r_1,r_2) \end{aligned}$$

Here the variable $r_k$ parametrizes the crossing point on the curve $\ell _k$ and we have expressed the average by means of the stationary probability density of the two intervals and the initial and final points on the starting and the target line, respectively^2^. Obviously, $r_1$ describes the final point for $I_1$ but also the initial point for $I_2$, and for the Markov process considered it is exactly this value $r_1$ that can carry memory between the intervals $I_1$ and $I_2$ (and also between $I_1$ and any higher interval $I_{1+k}$ with $k>1$). If the interspike interval sequence shows correlations, this is exclusively due to the fact that the final point of the first interval coincides with the initial point to the subsequent interval. If we choose the phase curve in such a way that the expected interval is always the same irrespective of the starting point, we eliminate the source of (linear) correlations— this is why we expect that correlations will vanish for a sequence of interphase intervals. In what follows we underpin this intuitive argument with a calculation.

Because of the Markov property of the stochastic process $\mathbf {x}(t)$, $I_2$ will depend only on the initial point $r_1$ but not on the previous initial point $r_0$. Based on this property, we can simplify the probability density as follows

8 $$\begin{aligned}&P(I_1,I_2,r_0, r_1,r_2)\nonumber \\&\quad =P(I_2|I_1,r_0,r_1,r_2)P(I_1,r_0, r_1,r_2)\nonumber \\&\quad =P(I_2|r_1,r_2)P(r_2|I_1,r_0, r_1)P(I_1,r_0, r_1)\nonumber \\&\quad =P(I_2|r_1,r_2)P(r_2|r_1)P(I_1|r_0,r_1)P(r_1|r_0)P(r_0) \end{aligned}$$

Here we have systematically split up multivariate probability densities into conditional densities and lower-dimensional multivariate densities (according to the scheme $P(x,y)=P(y|x)P(x)$) and have then used the Markov property to reduce the number of conditions. The conditional probability density for the second interval

$P(I_2|I_1,r_0,r_1,r_2)$ does neither depend on the first interval $I_1$ nor on the initial point of the first interval, $r_0$ and thus we can replace this by the conditional probability density $P(I_2|r_1,r_2)$, which has fewer arguments. Similarly, the statistics of the second target point $r_2$ does not depend on the first interval and its initial point $r_0$ and this is why $P(r_2|I_1,r_0, r_1)$ reduces to $P(r_2| r_1)$, etc.

Inserting (8) into (7), we can write the averaged product as follows:

9 $$\begin{aligned} \left\langle I_1I_2 \right\rangle= & {} \int dr_0 P(r_0) \nonumber \\&\times \int dr_1 \int dI_1 I_1 P(I_1|r_0,r_1)P(r_1|r_0)\nonumber \\&\times \int dr_2 \int dI_2 I_2 P(I_2|r_1,r_2)P(r_2|r_1) \end{aligned}$$

We emphasize that this holds true for any phase line. If we specifically use the MRT phase line (switching from the *I* notation to the *T* notation), the conditional mean value of the return time becomes independent of the initial point on $\ell$ (this was the defining feature of this line), and we obtain :

10 $$\begin{aligned} \int dr_2 \int dT_2 T_2 P(T_2|r_1,r_2)P(r_2|r_1)=\left\langle T \right\rangle . \end{aligned}$$

If we use this relation above, we can furthermore simplify the second set of integrals:

11 $$\begin{aligned} \int dr_1 \int dT_1 T_1 P(T_1|r_0,r_1)P(r_1|r_0)=\left\langle T \right\rangle . \end{aligned}$$

With this, the above relation reduces to

12 $$\begin{aligned} \left\langle T_1 T_2 \right\rangle =\left\langle T \right\rangle ^2 \int dr_0 P(r_0)=\left\langle T \right\rangle ^2. \end{aligned}$$

This, of course, corresponds to a vanishing covariance and, consequently, a vanishing first correlation coefficient, $\rho _1=0$. The above line of arguments can be repeated for intervals $T_1$ and $T_{1+k}$ with $k>1$, and thus, we expect that linear correlations vanish at all lags, i.e., $\rho _k=0 \; \forall k>0$. Finally, we note that our argument does not exclude that *nonlinear correlations among the intervals* can still exist—our derivation applies only to the linear correlations $\left\langle T_1 T_2 \right\rangle -\left\langle T_1 \right\rangle \left\langle T_2 \right\rangle$ but could not be extended, for instance, to the variances of the intervals because these follow a different phase line (as shown by Holzhausen et al. (2022) for one example).

## Examples of stochastic oscillators

### A planar oscillator with two stable limit cycles

We start with an example of a white-noise-driven isotropic (rotationally symmetric) planar oscillator, the so-called Guckenheimer–Schwabedal–Pikovsky oscillator (Guckenheimer 1975; Schwabedal and Pikovsky 2013)

13 $$\begin{aligned} {\dot{\rho }}= & {} g(\rho ) + \sigma \, \rho \, \xi _{\rho }(t)\nonumber \\ {\dot{\phi }}= & {} f(\rho )\nonumber \\ g(\rho )= & {} \rho \, (1-\rho )(3-\rho )(c-\rho ) + \sigma ^{2}\, \rho / 2,\nonumber \\ f(\rho )= & {} \omega + \gamma \, (\rho - 2) - (1 - \rho )\, (3 - \rho ). \end{aligned}$$

**Fig. 2:**
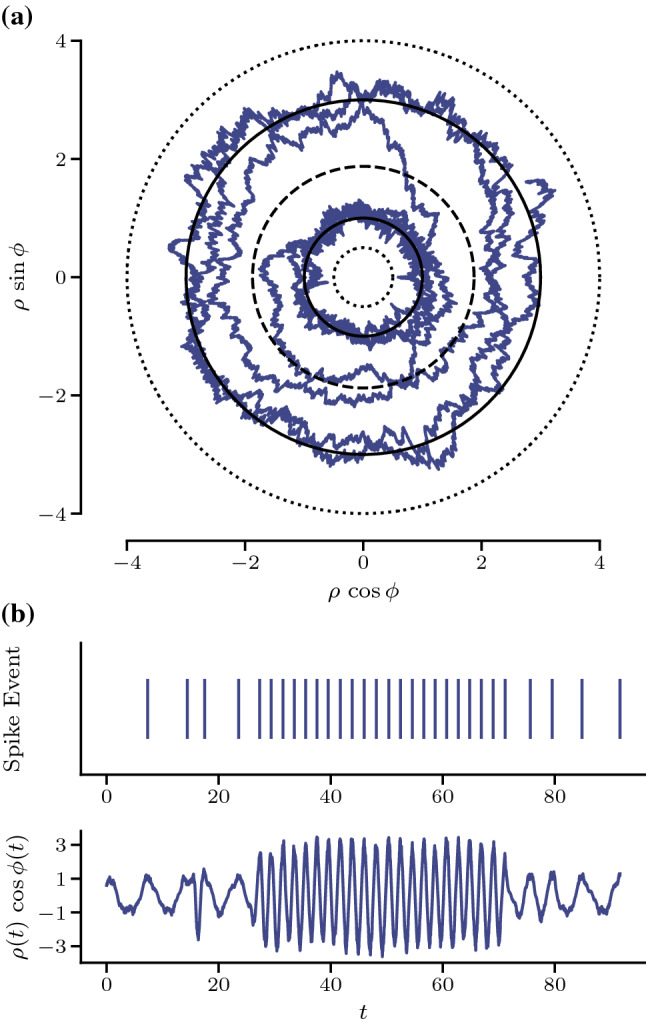
Guckenheimer–Schwabedal–Pikovsky oscillator. **a** A stochastic trajectory in Cartesian coordinates showing the switching between the two limit cycles (solid circles). **b** A spike sequence given by the crossing times of a spoke (top) and one of the components as a time series (bottom), revealing stochastic oscillations that are slower (for the inner limit cycle) and faster (for the outer limit cycle) leading to subsequences of shorter and longer intervals between spikes (see top panel)

This system shows stochastic transitions between the two stable limit cycles of the deterministic system at $\rho =1$ and $\rho =3$ when overcoming an unstable limit cycle at $\rho =c$ (with $1<c<3$); cf. Fig. 2a for an example trajectory in the phase space.

**Fig. 3:**
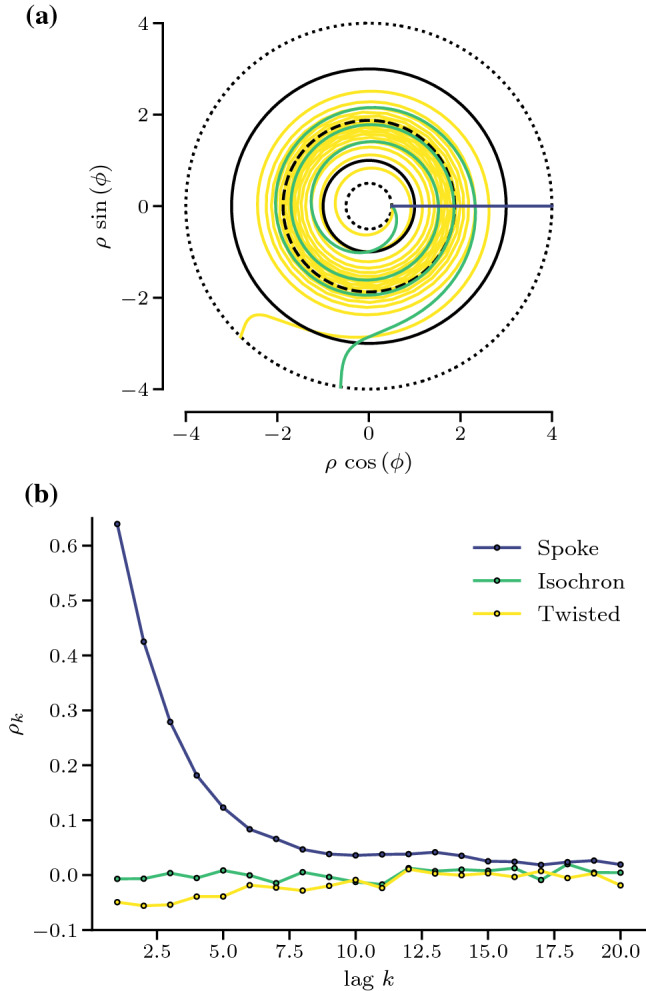
Event cross sections in phase space (**a**) and serial correlations of subsequent ISIs and IPIs (**b**) of the planar oscillator with two stable limit cycles (Guckenheimer–Schwabedal–Pikovsky oscillator). Stochastic periods $T_{i}$ are measured with respect to three event cross sections in phase space: a threshold line (spoke), MRT isochron *I* and the twisted MRT isochron $\phi _{I-\mathrm {Twisted}}(\rho ) = 5\, \phi _{I}(\rho )$. The black dotted line indicates the reflecting boundaries of the annulus domain at $\rho _{-}=0.5$ and $\rho _{+}=4.0$. Model parameters: $\sigma =0.37$, $\omega =2.0$, $\delta =1.0$, $c=1.875$. Numerical simulations of the model were performed using an explicit Euler–Maruyama scheme with time step $\varDelta t = 10^{-4}$

If we count rotations in a simple manner by first upcrossings of $\phi =N 2\pi$ (here *N* would be the rotation count or winding number), we obtain a sequence of stochastic intervals $I_i$ that is clearly positively correlated (see Fig. 3). Why do we see positive correlations? In simple terms, the speed is different on the two limit cycles—the difference is determined by the parameter $\gamma$ in (13). Consequently, the ISIs on one of the limit cycles will be on average different to the one on the other limit cycle, and both will deviate from the mean ISI. If transitions between the two limit cycles are not too frequent, we will see a number of shorter ISIs belonging to the outer limit cycle followed by a subsequence of longer intervals belonging to the inner limit cycle. Put differently, adjacent intervals deviate in the same manner from the mean interval which corresponds to positive interval correlations. The mechanism is also illustrated in Fig. 2b: only one Cartesian component of the oscillator is shown here, clearly elucidating the difference in oscillation frequency and the resulting subsequences of adjacent intervals that are all shorter or all longer than the mean ISI. Indeed, as becomes evident in Fig. 3b, counting intervals by the passages through the spoke of a wheel leads to pronounced positive ISI correlations.

The correlation lag is roughly given by the number of intervals it takes on average to switch between the limit cycles. The observed correlation could be analytically described by a theory that assumes Markovian switching of the firing between two rates and coefficients of variation (see Schwalger et al. (2012)).

For the system at hand, some of us have recently derived an analytical expression for the MRT phase in the form of a parametrization of the isochron (Holzhausen et al. 2022):

14 $$\begin{aligned} \phi _{\text {I}}(\rho ) = 2\int \limits _{\rho _{-}}^{\rho }\! dq\! \int \limits _{\rho _{-}}^{q} du \frac{f(u) - {\overline{\omega }}}{\sigma ^{2} u^{2}} \exp \left[ {-2 \int _{u}^{q} dv \frac{g(v)}{\sigma ^{2} v^{2}}}\right] .\nonumber \\ \end{aligned}$$

Here the mean rotation frequency (or, equivalently, the inverse of the mean rotation period) can also be calculated via (Holzhausen et al. 2022)

15 $$\begin{aligned} {\overline{\omega }} = \frac{2\pi }{{\overline{T}}}= \frac{\int _{\rho _{-}}^{\rho _{+}} d\rho \, f(\rho ) \, e^{-2 \int _{\rho }^{\rho _{+}} d\rho ^{\prime }\, g(\rho ^{\prime })/(\sigma \rho ^{\prime })^{2}}/ (\sigma \rho )^{2}}{\int _{\rho _{-}}^{\rho _{+}}d\rho \, e^{-2 \int _{\rho }^{\rho _{+}} d\rho ^{\prime }\, g(\rho ^{\prime })/(\sigma \rho ^{\prime })^{2}}/(\sigma \rho )^{2}}.\nonumber \\ \end{aligned}$$

We can now use this isochron to count rotations and create a sequence of IPIs. If we measure their SCC, all linear correlations are gone (see green line in Fig. 3b): $\rho _k\equiv 0$ for all $k>0$ in line with what we argued in Sect. 2.3. The isochron in this case is not a straight line between the inner and the outer boundary but it winds several times around the origin. This gives inner-laying points ($\rho <c$) of the same phase more head start compared to the faster moving points close to the outer limit cycle ($\rho >c$) that move with higher mean speed. And that is also the reason that the cause of positive correlations is now absent because the different speeds close to the outer and inner limit cycles are now compensated by the different starting points.

We also note that the sequence of IPIs is significantly *more irregular* than the ISIs. Below in the discussion section we uncover the general mechanism, why the CV should increase (decrease) when positive (negative) correlations are removed.

Last but not least we report an interesting finding for a counting curve that is five times as twisted as the isochron (here we have used $\phi (\rho ) = 5 \phi _I(\rho )$).In this case, correlations between the respective intervals become slightly negative. This illustrates that for a Markov process, the geometry of the counting line for the spikes or events controls the correlation of the intervals. Choosing the MRT isochron as counting line leads to vanishing correlations but in principle both positive and negative correlations are possible.

### An integrate-and-fire model with a spike-triggered adaptation current

We turn now to a simple yet very successful neuron model, the leaky integrate-and-fire (IF) model with an adaptation current (Treves 1993; Liu and Wang 2001; Chacron et al. 2001; Benda and Herz 2003) endowed with white Gaussian current noise (Schwalger and Lindner 2013). The equations of this system are as follows

16 $$\begin{aligned} \begin{aligned} {\dot{v}}&= \mu - v - a + \sqrt{2D}\xi (t), \\ \tau _a {\dot{a}}&= -a + \tau _a \varDelta _a \sum _i \delta (t - t_i) \end{aligned} \end{aligned}$$

with an additional fire-and-reset rule: Whenever the voltage variable reaches a certain threshold $v(t) = v_T$ a spike is fired at time $t_i = t$, at the same time *v*(*t*) is reset to some reset value $v_R$. In contrast, the adaptation variable *a* is *increased* by $\varDelta _a$ whenever a spike is fired. This does not require an additional reset rule but is incorporated directly into the dynamics of the adaptation variable by a sum over the delta functions. (The sum runs over the spike times of the IF model.) Further parameters are the mean input $\mu$ and the noise intensity *D* of the Gaussian white noise $\xi (t)$, that obeys the autocorrelation function $\langle \xi (t)\xi (t')\rangle = \delta (t-t')$. Here, we choose $\mu$ such that the deterministic neuron model ($D=0$) is mean-driven, i.e., there is no stable fixed point between $v_R$ and $v_T$, and even in the absence of noise the neuron fires repetitively.

**Fig. 4:**
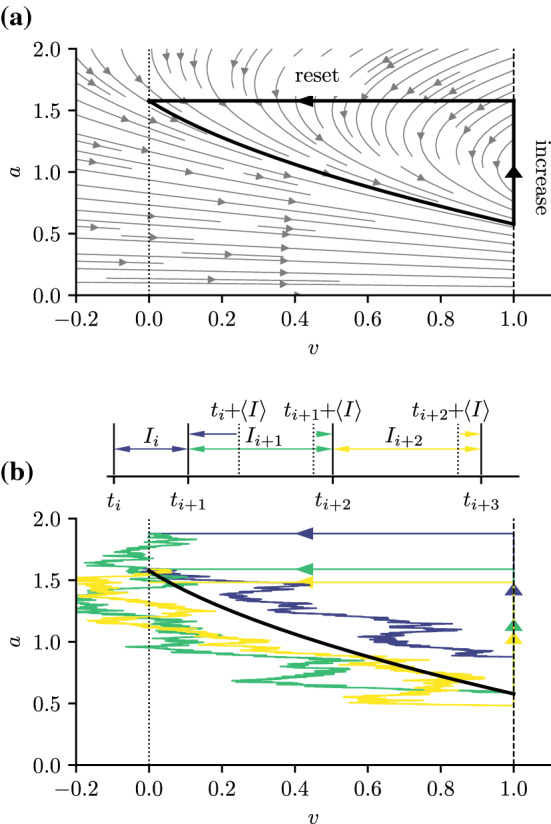
The adaptive leaky integrate-and-fire model as a planar oscillator. Panel (**a**) shows the deterministic vector field according to (16). The limit cycle (including increase and reset) is represented by the thick black line. The reset ($v_R = 0$) and threshold ($v_T = 1$) are shown by  dotted and dashed vertical lines, respectively. Panel (**b**) shows three stochastic trajectories with corresponding ISIs $I_{n} = t_{n+1} - t_{n}$ and spike times $t_{n}$. Dotted vertical lines in the upper panel indicate the times at which a spike would have been expected in the renewal case given that there has been a spike at $t_n$, i.e., at $t_n + \langle I \rangle$. Because the first ISI $I_i$ is shorter than the average interval ($t_{i+1} < t_{i} + \langle I \rangle$) the following intervals $I_{i+k}$ with $k=1,2$ are more likely to be prolonged ($t_{i+k} > t_{i+k-1} + \langle I \rangle , \, k=1,2$) indicating negative interval correlations over several lags *k*. Parameters (a, b): $\mu =2$, $\tau _a=2$, $\varDelta _a = 1$ and $D=(0, 0.1)$

We can interpret this model as a two-dimensional oscillator, with the caveat that a certain part of the plane is cut out and the dynamics in this cutout part are replaced by the fire-and-reset condition—it is exactly the stereotypical shape of the action potential that is not modeled in an integrate-and-fire framework. We can still think of the deterministic dynamics of the model for $D=0$ as governed by a limit cycle (Schwalger et al. 2010; Schwalger and Lindner 2013). This limit cycle is represented by a thick black line in Fig. 4a: It extends only from the reset line $v_R=0$ to the threshold line $v_T=1$ and includes two infinitely fast parts, the increase of the adaptation variable by $\varDelta _a$ and the reset to the reset voltage $v_R$. Indeed, in the deterministic system, all initial values will lead to a trajectory close to the limit cycle.

**Fig. 5:**
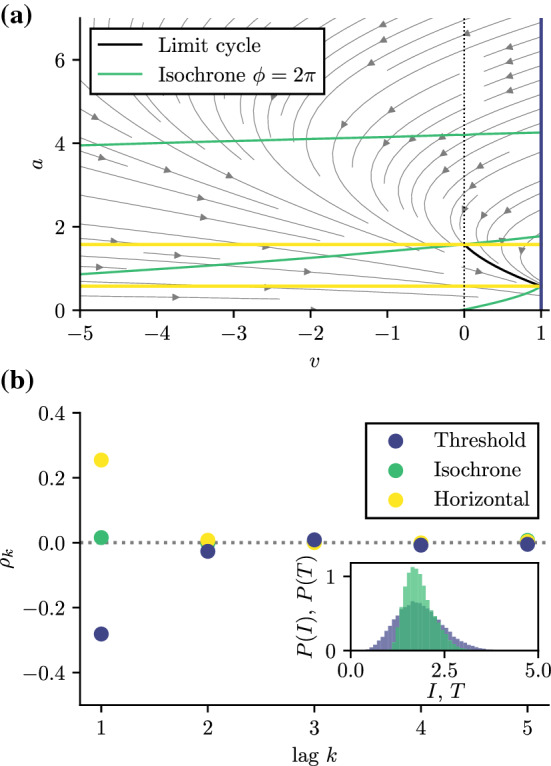
Isochron and interval correlations for the adaptive leaky integrate-and-fire model. **a**: Deterministic vector field including limit cycle, reset, and threshold (dark blue line) but over a larger domain compared to Fig. 4a and including the deterministic isochron with phase $\phi = 2\pi$ (green line) and the horizontal (yellow) line as another (rather arbitrary) example of how a spike sequence could be defined ($l_n = \{(v, a) \mid a = a_{0} + n\varDelta _a, v \in [v_{\mathrm{min}} , v_T] \}\}$ with vertical spacing $\varDelta _a$ and $a_0$ being the adaption variable of the limit cycle at the threshold). Because the phase of the isochron was chosen to be $\phi = 2\pi$ the isochron passes the limit cycle right at the threshold and accordingly at the reset point, which corresponds to $\phi =0$. **b**: Interval correlations where the intervals are defined as the time between the successive crossing of the threshold, isochron, or certain horizontal lines, as shown in **a**. Serial correlations of the ISI (blue circles) are negatively correlated; IPI correlations (green circles) defined by the crossings of the isochrons vanish as expected; intervals defined by subsequent crossings of the horizontal lines are positively correlated (yellow circles). Parameters (a, b): $\mu =2$, $\tau _a=2$, $\varDelta _a = 1$ and $D=(0, 0.1)$

The standard way of counting spikes and generating a sequence of ISIs is the passage of the voltage threshold; equivalently, we can think of the reset events as forming a point process. The ISIs are typically negatively correlated (see blue circles in Fig. 5b) as is well known from the theoretical literature (Liu and Wang 2001; Chacron et al. 2000; Schwalger et al. 2010; Schwalger and Lindner 2013; Shiau et al. 2015) and also from experimental recordings (see reviews by Farkhooi et al. (2009); Avila-Akerberg and Chacron (2011)).

We consider here a case of weak adaptation, for which the SCC is negative at all lags (Schwalger and Lindner 2013). Why are correlations between adjacent intervals negative? In Fig. 4 we have depicted three successive interspike intervals (b, top) together with their stochastic trajectories (b, bottom). The first trajectory (dark blue) starts close to the limit cycle and reaches the threshold quickly. This can either be seen from the top of panel (b) where $I_i$ is much shorter than the mean interval $I_i < \langle I \rangle$ or from the bottom of panel (b) where the trajectory crosses the threshold above the limit cycle (even though it started close to the limit cycle). The latter is related to the length of the interval because the dynamics of the adaptation imply a simple exponential decay over the course of an ISI. Hence, if we find a larger value of the adaptation variable at the end of an ISI (compared to the limit cycle) that is because this ISI was shorter than the mean ISI. Now consider the second interval (green). The initial condition of the adaptation variable for this trajectory is determined by the length of the previous interval. In particular, since the first interval was shorter than the mean, the initial value of *a* for the second interval will be larger than on average (see Fig. 4b—the green trajectory starts above the limit cycle). From (16) it becomes evident that an increase in *a* will slow down the *v* dynamics. The second trajectory will thus, again on average, reach the threshold after some time that is larger than the mean ISI. This can be seen in the top part of panel (b), where the second interval is indeed prolonged $I_{i+1} > \langle I \rangle$. A weaker version of the same effect still applies to the third trajectory (yellow), i.e., the trajectory still starts slightly above the limit cycle. To summarize, an initial, shortened interval leads to an increase of the adaptation variable that prolongs the subsequent intervals—this is the mechanism by which all subsequent intervals are negatively correlated with the first interval.

In line with the above explanation, we expect that these serial correlations vanish for the IPIs, i.e., the time between successive crossings of the MRT-isochron. Even the deterministic definition of the phase ($D=0$) has not been studied for this model to the best of our knowledge. For simplicity, we restrict ourselves in the following to the deterministic isochron that, for weak noise and in the mean-driven regime, we assume to be a good approximation for the MRT-isochron; we have extracted the phase isochron for the deterministic system as outlined in the appendix Sect. A.

The resulting isochron for one specific phase $\phi =2\pi$ is shown in Fig. 5a; as can be seen, there are several branches that belong to the same phase. This is a consequence of the reset rule, which, in the simplest case, can be understood as follows: Consider a point on the isochron that lies directly on the threshold $p_T = (v_T, a(v_T))$; due to the reset rule, a trajectory that starts at $p_T$ will be reset to $p_R = (v_R, a(v_T) + \varDelta _a)$ (see Fig. 8d). This reset does not take any time; therefore, the return time to the isochron starting at $p_R$ or $p_T$ is the same and both points should belong to the same isochron. This argument is valid with one restriction: The deterministic system has to pass the threshold at $p_T$, i.e., ${\dot{v}}(p_T) > 0$ must hold true.

If we now count rotations for a weakly stochastic adaptive integrate-and-fire model by the passage of a (deterministic) isochron, we can construct a sequence of IPIs, for which the SCC vanishes at all lags *k* to a very good approximation (see Fig. 5b). Also the standard deviation of the IPIs is significantly smaller than that of the ISIs as can be seen from the distributions of the two types of intervals (see inset in Fig. 5b). Hence, for weak noise we confirm again our general result derived in Sect. 2.3.

Interestingly, if we use an alternative phase definition that is very different from both the constant voltage or the deterministic isochron, namely, a set of horizontal lines (constant adaptation, yellow in Fig. 5a), for counting rotations, the serial correlation coefficient becomes *positive* (cf. yellow circles in Fig. 5b; additional features of the interval’s probability density are discussed in the appendix, Sect. B). This is yet another example for how the geometry of the counting lines determines the correlations of the corresponding interval sequences.

### A Hodgkin–Huxley model with channel noise

As our last example, we consider the classical Hodgkin–Huxley model endowed with channel noise. Following Skaugen and Walløe (1979), at the molecular level we take the sodium channel to comprise three independent binary “m” gates and one independent binary “h” gate, leading to a channel state graph with eight vertices and 20 directed edges. Similarly, we take the potassium channel to comprise four independent binary “n” gates, leading to a channel state graph with five vertices and eight directed edges. See Fig. 10 in appendix C for illustration. Given a total population of $M_\text {tot}$ sodium and $N_\text {tot}$ potassium channels, we define the state vectors

17 $$\begin{aligned} {\mathbf {M}}&=[M_{00},M_{10},M_{20},M_{30},M_{01},M_{11},M_{21},M_{31}]^\intercal \nonumber \\&\quad \in [0,1]^{8} \end{aligned}$$

18 $$\begin{aligned} {\mathbf {N}}&=[N_0,N_1,N_2,N_3,N_4]^\intercal \in [0,1]^5, \end{aligned}$$

each summing to unity. The net sodium conductance is $M_{31}$ (the fraction of sodium channels in the open state) multiplied by ${\overline{g}}_\text {Na}$ (the maximal sodium conductance); the net potassium conductance is ${\overline{g}}_\text {K}N_4$. Each of the 28 directed edges in Fig. 10 represents a particular channel state transition, i.e., opening or closing a single gate. We take each such edge to be an independent source of fluctuations. In the large channel population limit, the resulting diffusion approximation (Fox and Lu 1994; Goldwyn and Shea-Brown 2011; Goldwyn et al. 2011) gives a system obeying the following set of Langevin equations (Pu and Thomas 2020, 2021):

19 $$\begin{aligned} C\frac{dV}{dt}= & {} -{\bar{g}}_{\text {Na}}M_{31}(V-V_{\text {Na}})-{\bar{g}}_{\text {K}}N_4(V-V_\text {K})\nonumber \\&\quad -g_\text {L}(V-V_\text {L})+I_\text {app}, \end{aligned}$$

20 $$\begin{aligned} \frac{d{\mathbf {M}}}{dt}= & {} A_\text {Na}(V){\mathbf {M}}+S_\text {Na}\xi _\text {Na}(t), \end{aligned}$$

21 $$\begin{aligned} \frac{d{\mathbf {N}}}{dt}= & {} A_\text {K}(V){\mathbf {N}}+S_\text {K}\xi _\text {K}(t). \end{aligned}$$

Here, *C* ($\mu \text {F/cm}^2$) is the capacitance, $I_\text {app}$ ($\text {nA/cm}^2$) is the applied current, the maximal conductance is ${\bar{g}}_\text {ion}$ ($\text {mS/cm}^2$), $V_\text {ion}$ (mV) is the associated reversal potential, for $\text {ion}\in \{\text {Na}^+,\text {K}^+\}$, and the Ohmic leak current is $g_\text {leak}(V-V_\text {leak})$. The voltage-dependent drift matrices, $A_\text {Na}$ ($8\times 8$) and $A_\text {K}$ ($5\times 5$), and the $8\times 20$ $\text {Na}^+$ noise coefficient matrix $S_\text {Na}$, and the $5\times 8$ matrix $S_\text {K}$, are derived by Pu and Thomas (2020, 2021) and reproduced in Appendix C.

For this 14-dimensional HH system we extract a sequence of interspike intervals (ISIs) and interphase intervals (IPIs) as follows.

In order to find the sequence of voltage spikes, we set a threshold voltage of $V_\text {th}=-20$ mV. Each spike time is determined as the upcrossing time of $V_\text {th}$. Because all noise in the model is contained in the gating variables, rather than the voltage, the voltage is continuously differentiable and there is no ambiguity about the spike times.

In order to find the interphase interval sequence, we track the times at which the simulated trajectory crosses the deterministic isochron that passes through the deterministic limit cycle trajectory at $V=V_\text {th}$. Cao et al. provided a method for calculating the MRT isochrons for planar systems, but the method does not readily extend to a 14-dimensional phase space. However, in the small noise regime, we assume that the MRT isochron is close to the classical deterministic limit cycle isochron, which we use as an approximation. Thus we track the phase of the trajectory and mark one isophase crossing every time the phase advances by $2\pi$. See Appendix C for details.

If we simulate the system for a large number of channels (implying a weak noise intensity) and measure spike times and corresponding ISIs by upcrossings of a voltage threshold, we observe a weak but significantly negative correlation ${\bar{\rho }}_1=-0.0226 \pm 8.9194e\text {-}04$ (mean ± standard error of the mean (SEM)). Here we simulate the stochastic HH model using the same framework as Eq. 3 by Pu and Thomas (2021), and set $\epsilon =0.0281$. The mean and standard deviation are calculated from 400 simulations, where each single simulation contains more than 10,200 ISIs. If we measure IPIs using the deterministic phase (which for weak noise should be rather close to the stochastic MRT phase), we get a correlation coefficient at lag one of ${\bar{\rho }}_1=-0.0012 \pm 8.9317e\text {-}04$. We applied the one-sample *t*-test to test the null hypothesis that $\rho _1(\text {ISI})$ has a mean zero at the 5% significance level (and $\rho _1(\text {IPI})$ similarly). The test result rejects the null hypothesis for the ISIs, with a *p*-value $=5.9797e\text {-}85$, and accepts the null hypothesis for the IPIs, with a *p*-value $=0.1927$. Hence, as for the other two systems we can confirm our general result.

**Fig. 6:**
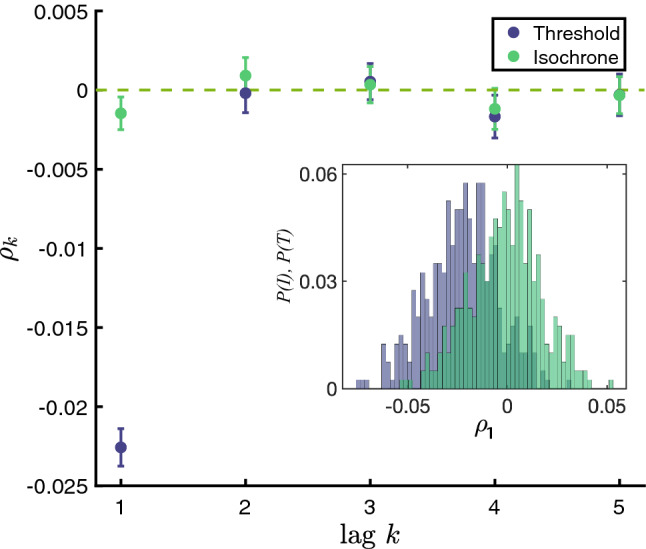
Negative ISI correlations in the stochastic Hodgkin–Huxley model and vanishing correlations for the associated IPI sequence. The inset shows the statistical distribution of $\rho _1$ for the ISIs (in light blue) and IPIs (in green) based on an ensemble of 400 trials (each containing more than 10,200 oscillations). Error bars indicate the mean ± SEM

**Fig. 7:**
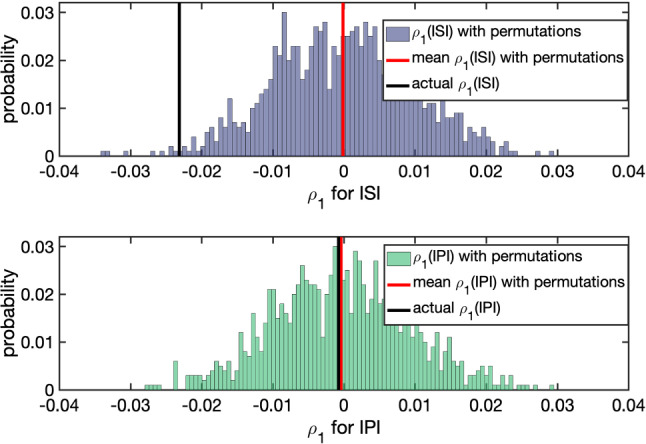
Validating the significance of negative serial correlation coefficient. Each histogram plots the distribution of $\rho _1$ for 1000 randomly shuffled sequences of ISIs (top) or IPIs (bottom). Black bar: $\rho _1$ of the unshuffled sequence. Red: mean $\rho _1$ of the shuffled sequences

To further illustrate the significance or insignificance of the negative correlation coefficient at lag one, we compare the ISI and IPI statistics to that of the corresponding sequences of shuffled intervals. We recorded $\rho _1$ for each permutation and plotted a histogram. Figure 7 presents an example of the distributions of $\rho _1$ for ISIs and IPIs with 1000 randomly permutations. The mean($\rho _1$) (in red) is the mean of the $\rho _1$ values, obtained from the 1000 permutations, which are almost 0 in both cases. Mean$(\rho _1(\text {ISI}))=-1.4626e\text {-}04$ and mean$(\rho _1(\text {IPI}))=-4.1438e\text {-}04$. The actual $\rho _1$’s of the original (unshuffled) spike trains are plotted in a black bar, where $\rho _1(\text {ISI})=-0.0232$ and $\rho _1(\text {IPI})=-7.7171e\text {-}04$. The one-sample *t*-test suggested to accept the null hypothesis that “mean($\rho _1(\text {ISI})$) (and mean($\rho _1(\text {IPI})$)) has a mean zero” at the 5% significance level, with a *p*-value of 0.6357 for ISIs and 0.1731 for IPIs. Given the distributions of the $\rho _1(\text {ISI})$ with permutations, the z-score of observing $\rho _1(\text {ISI})=-0.0232$ is $-1.6989e03$, whose probability is almost zero. For the IPIs, the z-score of observing $\rho _1(\text {IPI})=-7.7171e\text {-}04$ is -1.0851, where we are in favor of the null hypothesis that $\rho _1(\text {IPI})$ is from the distribution of $\rho _1$ for the shuffled IPIs.

## Discussion and conclusions

We have found an interesting property of the recently introduced MRT phase in multidimensional oscillator models: Rotation counts of these systems form in general a non-renewal point process if standard threshold criteria are used; however, if the isochron of the MRT phase is used, at least linear correlations would vanish. This finding has been mathematically derived above but can also be intuitively understood as follows. For a Markov process correlations between adjacent passage intervals can arise only due to their shared point in space (which is the final point of the first interval and the initial point for the second interval). The correlation between the intervals can be regarded as a conditional mean value of the second interval, but if this mean value becomes independent of the initial point in space (the point on the MRT phase line), it becomes independent of the first interval.

Counting according to the MRT phase gives us thus an (approximate) renewal process, which is a great simplification because for these processes many formulas for their basic statistics and relationships between different statistics are known (Cox 1962). It might even be possible to use this mapping (from the model’s phase space to the MRT phase) to find novel ways to calculate the serial correlation for the standard threshold counting, although we have to admit at this point that we have not yet an idea how to practically do this.

One motivation for the calculation of the interspike interval’s correlation coefficient is the effect that $\rho _k$ has on the long-term variability of the spike train. In particular, the long-term asymptotics of the Fano factor of the counting process is given by

22 $$\begin{aligned} \lim \limits _{T\rightarrow \infty } F(T)= & {} \lim \limits _{T\rightarrow \infty } \left\langle (N(T)-\left\langle N(T) \right\rangle )^2 \right\rangle /\left\langle N(T) \right\rangle \nonumber \\= & {} C_V^2 (1+2\sum _{k=1}^\infty \rho _k). \end{aligned}$$

Hence, purely negative correlations over all lags, for instance, are known to reduce the Fano factor while positive correlations will increase it. The long-term Fano factor is also intimately related to the spike-train power spectrum via the relation (Cox and Lewis 1966)

23 $$\begin{aligned} \lim \limits _{f\rightarrow 0} S(f)=r_0 \lim \limits _{T\rightarrow \infty } F(T)=r_0C_V^2 (1+2\sum _{k=1}^\infty \rho _k), \end{aligned}$$

where $r_0$ is the firing rate of the neuron (the inverse of the mean ISI). Negative correlations, for instance, can lead to a considerable drop of power at low frequencies while positive correlations boost the spectrum in this range. These effects of correlations on the spontaneous power spectrum can be relevant for the transmission of weak time-dependent signals in the neural spike train (Chacron et al. 2004; Lindner et al. 2005; Blankenburg and Lindner 2016), because the spontaneous spectrum (the spectrum in the absence of a stimulus) serves as the background spectrum in the presence of a stimulus and may affect the signal-to-noise ratio.

Does this mean that with the removal of negative correlations in the neuron model with adaptation, we have removed the potentially beneficial effect as well? We do not think that this is the case for the following reason. The long-term statistics of the count will not depend on the exact way we count *phase rotations* or *spikes* as long as we do not leave out events or introduce new ones. Hence, we expect that irrespective of the way we count rotations, the long-term values of the count’s mean and variance is always the same and, consequently, we have the same Fano factor in all cases, in particular:

24 $$\begin{aligned} F_{\text {ISI}}=F_{\text {IPI}} \end{aligned}$$

and thus we have

25 $$\begin{aligned} C_{V,\text {ISI}}^2\left( 1+2\sum _{k=1}^\infty \rho _{k,ISI}\right) =C_{V,\text {IPI}}^2. \end{aligned}$$

In all our examples, we have checked this relation numerically and confirmed it. It also concisely explains why a renewalization of the spike train of an adapting neuron comes along with a reduction of the CV, while in the case of a system with bistable behavior and positive ISI correlations, the CV becomes larger when going over to the IPI sequence.

For the special case of an integrate-and-fire dynamics with an adaptation current, the non-renewal dynamics has been related by Nesse et al. (2010, 2021) in another way to the variations of an independent (renewal-like) variable, the increments in the adaptation variable. The authors of these studies also speculate how this independent variable might be read out by a postsynaptic readout neuron via matched synaptic kinetics. Whether the relation to the increments of the adaptation variable is somehow related to our mapping to the MRT phase and the approximated *renewalization* is unclear at the moment but certainly worth further exploration. Likewise, it would be interesting how the phase concept and the vanishing of the correlation coefficient apply to generalized models of adapting neurons, for instance, models with subthreshold adaptation components (Shiau et al. 2015) or correlated Gaussian noise (Ramlow and Lindner 2021).

## Figures and Tables

**Fig. 8:**
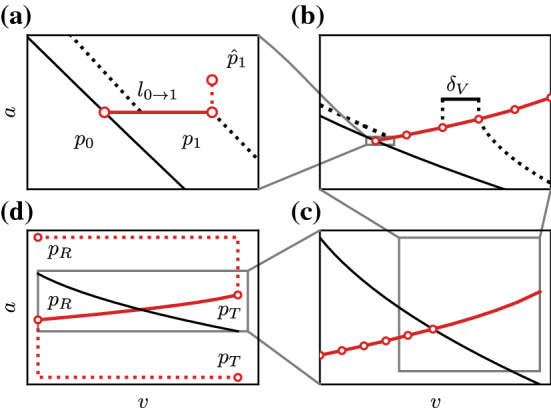
Numerical procedure to determine the deterministic isochron. For a detailed description see the main text of Sec. A. Parameters: $\mu =2$, $\tau _a=2$, $\varDelta _a = 1$ and $D=0$

**Fig. 9:**
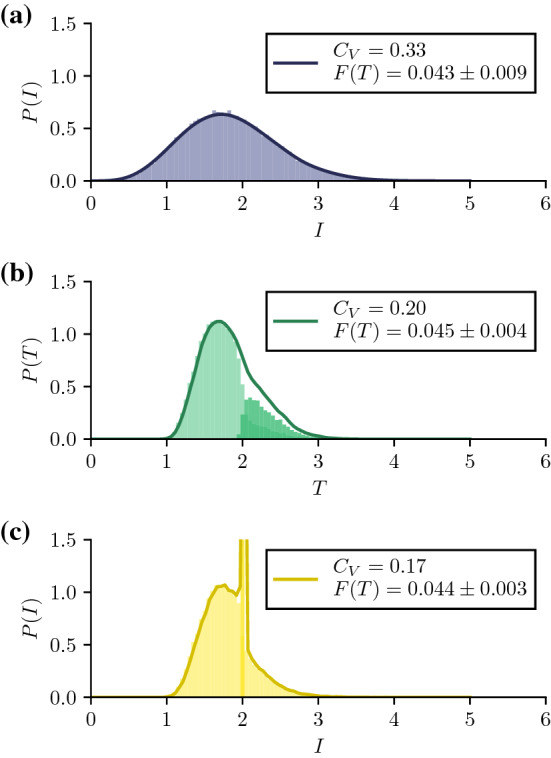
Probability density for different intervals. The interval distribution is shown by a solid line. For each panel the intervals are defined as the time between the successive crossings of a certain line. This curve is either the threshold (**a**), isochron (**b**) or a set of horizontal lines with vertical spacing $\varDelta _a$ (**c**), cf. Fig. 5. Interestingly, and most pronounced in the third case intervals with (dark) or without (bright) threshold crossing have distinct probability densities. Parameters: $\mu =2$, $\tau _a=2$, $\varDelta _a = 1$ and $D=0$

**Fig. 10:**
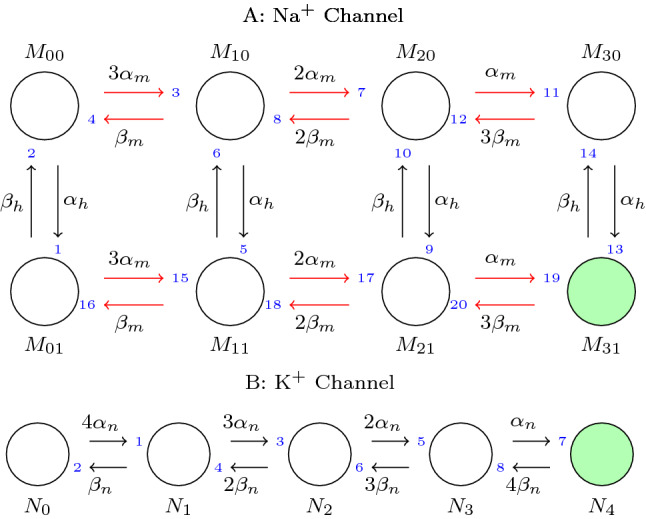
Transition state diagram for the stochastic Hodgkin–Huxley model, redrawn with permission from Pu and Thomas (2021). **A:** $\hbox {Na}^{+}$ states and transitions. **B:** $\hbox {K}^{+}$ states and transitions. States marked in green shading represent conducting states $M_{31}$ ($\hbox {Na}^{+}$) and $N_4$ ($\hbox {K}^{+}$). See App. C for voltage-dependent per capita transition rates for each directed edge ($\alpha _n$, $\beta _n$, $\alpha _m$, $\beta _m$, $\alpha _h$ and $\beta _h$) Small blue numerals label the directed edges 1-8 ($\hbox {K}^{+}$ channel) and 1-20 ($\hbox {Na}^{+}$-channel). Transitions involving the $\hbox {Na}^{+}$ channel fast activation (m) gates are marked with red arrows

## Numerical procedure for the deterministic isochron of the leaky integrate-and-fire model with adaptation

We assume that the deterministic system approaches for all initial conditions a limit cycle with period *T*. To construct the isochron we proceed as follows. First, we choose a point on the limit cycle $p_{0} = (v_{0}, a_{0})$ that serves as a starting point for the construction of the corresponding isochron. The phase of that point can easily be found using that i) the phase on the limit cycle at the reset and threshold voltages are $\phi =0$ and $\phi = 2\pi$, respectively; ii) the phase evolves with constant velocity ${\dot{\phi }} = 2\pi /T$ (sometimes ${\dot{\phi }} = 1/T$ if $\phi \in [0, 1]$).

Second, we construct the isochron above (right) and below (left) the limit cycle separately. Starting above, we choose a point that is *off* the limit cycle (not yet necessarily on the isochron) $p_1 = (v_1, a_1$) and define a straight-line segment

$$\begin{aligned} l_{0 \rightarrow 1} = \{(v, a) \mid a = a_{0} + \frac{a_1 - a_{0}}{v_1 - v_{0}}(v - v_{0}), v \in [v_0 , v_1] \}\} \end{aligned}$$

that connects $p_0$ and $p_1$ (see Fig. 8a). This segment will eventually become a local linear approximation of the isochron. Let $p_1$ evolve according to (16) with $D=0$ and measure the return time $\tau$ from $p_1$ to $l_{0\rightarrow 1}$. Note that it is guaranteed that the trajectory will pass $l_{0 \rightarrow 1}$ because the stability of the limit cycle implies that the trajectory gets closer to the limit cycle (for an exemplary trajectory starting at $p_1$ see Fig. 8a black dotted line). If $p_1$ is already on the isochron, the return time will exactly match the deterministic period $\tau = T$. If that is not yet the case the adaptation variable of $p_1 = (v_1, a_1)$ is adjusted as follows: If $\tau$ is smaller/larger than *T* the variable $a_1$ is increased/decreased by some value $\varepsilon _a$. This defines a new point ${\hat{p}}_1 = (v_1, a_1 \pm \epsilon _a)$ (see Fig. 8a) for which the aforementioned procedure (define a curve from $p_0$ to ${\hat{p}}_1$, measure the return time $\tau$, adjust $a_1$) is repeated until $\tau - T < \varepsilon _\tau$, i.e., until the return time is sufficiently close to the deterministic period. (As an error criterion we choose $\varepsilon _\tau = 10^{-3} T$.) We have now found one segment of the isochron, namely $l_{0\rightarrow 1}$.

Third, to find the whole isochron over a certain domain $[v_\mathrm{min}, v_T]$ the isochron has to be extended. To do so, the aforementioned procedure is in principle repeated for further points $p_{i} = (v_{i-1} \pm \delta _v, a_{i-1})$ where $p_{i-1} = (v_{i-1}, a_{i-1})$ is the previous point and $\delta _v$ is the *v*-spacing between adjacent points on the isochron (see Fig. 8b). The connecting segment $l_{i-1 \rightarrow i}$ is defined as before but the return time is measured with respect to the entire preliminary isochron

$$\begin{aligned} l_{0 \rightarrow i} = {\left\{ \begin{array}{ll} l_{0 \rightarrow 1}, \quad \text {for } v \in [v_0 , v_1], \\ l_{1 \rightarrow 2}, \quad \text {for } v \in [v_1 , v_2], \\ \vdots \\ l_{i-1 \rightarrow i}, \quad \text {for } v \in [v_{i-1}, v_{i}]. \end{array}\right. } \end{aligned}$$

The procedure is repeated for the part of the isochron that lies below the limit cycle (see Fig. 8c). Finally, due to the reset rule, the isochron has multiple branches. This was already explained in the main part. As a reminder if $p_T = (v_T, a(v_T))$ lies on the isochron so does $p_R = (v_R, a(v_T) + \varDelta _a)$ with the restriction that the deterministic system has to pass the threshold at $p_T$, i.e., ${\dot{v}}(p_T) > 0$ must hold true. Starting at $p_R$ the isochron can be constructed point-by-point as before.

## Features of the interval probability densities of the leaky integrate-and-fire model with adaptation

Here, we report an interesting finding for oscillators with a reset mechanism and a counting procedure that deviates from a simple voltage threshold passing. If the intervals are not counted with respect to the crossing of the threshold that defines the reset but some other line, we find that the intervals fall into two categories with distinct interval distribution: Intervals during which the threshold was crossed versus intervals during which the threshold was not crossed. The distributions for these two types of intervals can be seen in Fig. 9b, c where the darker distribution indicates that the threshold was crossed. The actual interval distribution, i.e., the sum of the two aforementioned distributions, is plotted as a solid line. The interval distribution for successive crossings of horizontal lines, shown in Fig. 9c, requires explanation. The distribution of the intervals that do not include a crossing of the threshold is a delta function, i.e., the length of the interval is fixed because the decay of the adaptation variable follows a completely deterministic dynamics. To be more specific, consider two horizontal lines $l_{n} = \{(v, a) \mid a = a_{0} + n\varDelta _a, v \in [v_{\mathrm{min}} , v_T] \}$ with fixed $a_0$ and $n=0,1$ and calculate the time it takes to get from $l_1$ to $l_0$. Because the dynamics of the adaptation variable without reset are deterministic, it will take exactly $t_{1\rightarrow 0}=\tau _a \log (1 + \varDelta _a/a_0)$ to reach $l_0$ given that the trajectory started at $l_1$. Therefore, the corresponding probability density is a delta function at $t_{1\rightarrow 0}$ and accounts for approximately $20\%$ of all intervals. Note that even though the probability *P*(*I*) includes a delta function the Fano factor is still preserved.

**Table 1:** Parameters used for stochastic Hodgkin–Huxley simulations

Symbol	Meaning	Value
*C*	Membrane capacitance	1 $\mu F/cm^2$
${\bar{g}}_\text {Na}$	Maximal sodium conductance	120 $\mu S/cm^2$
${\bar{g}}_\text {K}$	Maximal potassium conductance	36 $\mu S/cm^2$
$g_\text {leak}$	Leak conductance	0.3 $\mu S/cm^2$
$V_\text {Na}$	Sodium reversal potential for $\hbox {Na}^{+}$	50 *mV*
$V_\text {K}$	Potassium reversal potential for $\hbox {K}^{+}$	-77 *mV*
$V_\text {leak}$	Leak reversal potential	-54.4 *mV*
$I_\text {app}$	Applied current to the membrane	10 $nA/cm^2$
${\mathcal {A}}$	Membrane Area	$100\,\mu \text {m}^2$
$M_\text {tot}$	Total number of $\hbox {Na}^{+}$ channels	6,000
$N_\text {tot}$	Total number of$\hbox {K}^{+}$ channels	18,00

## Hodgkin–Huxley model details

### HH channel state transition graph and model parameters

In this appendix we provide details of the stochastic Hodgkin–Huxley Langevin model. Figure 10 illustrates the ion channel state transition diagram. Panel A shows eight distinct sodium channel states, ${\mathbf {M}}= [M_{00},M_{10},M_{20},M_{30}, M_{01},M_{11},M_{21},M_{31}]^\intercal$, connected by 20 directed edges. Red arrows indicate edges with fast transition kinetics (m-gate transitions). Panel B shows five distinct potassium channel states, ${\mathbf {N}}=[N_0,N_1,N_2,N_3,N_4]^\intercal$, connected by eight directed edges. Table 1 provides model parameters.

The subunit kinetics for Hodgkin and Huxley parameters are given by the following equations

26 $$\begin{aligned} \alpha _m(V)&= \frac{0.1*(25-V)}{ \exp (2.5-0.1V)-1} \quad&\beta _m(V)&= 4*\exp (-V/18) \end{aligned}$$

27 $$\begin{aligned} \alpha _h(V)&= 0.07*\exp (-V/20) \quad&\beta _h(V)&= \frac{1}{ \exp (3-0.1V)+1} \end{aligned}$$

28 $$\begin{aligned} \alpha _n(V)&= \frac{0.01* (10-V)}{\exp (1-0.1V)-1}\quad&\beta _n(V)&= 0.125\exp (-V/80) \end{aligned}$$

The mean field sodium transition rate matrix (cf. (20)) is given by:

29 $$\begin{aligned} A_\text {Na}(V) =\begin{bmatrix} A_\text {Na}(1) &{} \beta _m&{}0 &{}0 &{}\beta _h&{}0&{}0&{}0\\ 3\alpha _m&{}A_\text {Na}(2)&{}2\beta _m&{}0&{}0&{}\beta _h&{}0&{}0\\ 0&{}2\alpha _m&{}A_\text {Na}(3) &{}3\beta _m&{}0&{}0&{}\beta _h&{}0 \\ 0&{}0&{}\alpha _m&{}A_\text {Na}(4)&{}0&{}0&{}0&{}\beta _h \\ \alpha _h&{}0&{}0&{}0&{}A_\text {Na}(5)&{}\beta _m&{}0&{}0\\ 0&{}\alpha _h&{}0&{}0&{}3\alpha _m&{}A_\text {Na}(6)&{}2\beta _m&{}0\\ 0&{}0&{}\alpha _h&{}0&{}0&{}2\alpha _m&{}A_\text {Na}(7)&{}3\beta _m\\ 0&{}0&{}0&{}\alpha _h&{}0&{}0&{}\alpha _m&{}A_\text {Na}(8)\\ \end{bmatrix}, \end{aligned}$$

The mean field potassium transition rate matrix (cf. (21)) is given by:

30 $$\begin{aligned} A_\text {K}(V) =\begin{bmatrix} A_\text {K}(1)&{} \beta _n(V) &{} 0 &{} 0 &{} 0\\ 4\alpha _n(V)&{} A_\text {K}(2)&{} 2\beta _n(V) &{} 0&{} 0\\ 0&{} 3\alpha _n(V)&{} A_\text {K}(3)&{} 3\beta _n(V)&{} 0\\ 0&{} 0&{} 2\alpha _n(V)&{} A_\text {K}(4)&{} 4\beta _n(V)\\ 0&{} 0&{} 0&{} \alpha _n(V)&{} A_\text {K}(5) \end{bmatrix}, \nonumber \\ \end{aligned}$$

with diagonal elements

$$\begin{aligned} A_\text {ion}(i)=-\sum _{j\,:\,j\ne i}A_\text {ion}(j,i),\text { for ion }\in \{\text {Na},\text {K}\}. \end{aligned}$$

The noise coefficient matrices $S_\text {K}$ and $S_\text {Na}$ in (20)-(21) are given by

$$\begin{aligned} S_\text {K}=&\frac{1}{\sqrt{N_\text {ref}}}\left[ \begin{array}{cccccccc} -\sqrt{4\alpha _n n_0}&{} \sqrt{\beta _n n_1}&{}0&{}0&{}0&{}0&{}0&{}0\\ \sqrt{4\alpha _n n_0}&{} -\sqrt{\beta _n n_1}&{}-\sqrt{3\alpha _n n_1}&{}\sqrt{2\beta _n n_2}&{}0&{}0&{}0&{}0 \\ 0 &{}0 &{}\sqrt{3\alpha _n n_1}&{}-\sqrt{2\beta _n n_2}&{}-\sqrt{2\alpha _n n_2}&{}\sqrt{3\beta _n n_3}&{}0&{}0\\ 0 &{}0&{}0&{}0 &{} \sqrt{2\alpha _n n_2}&{}-\sqrt{3\beta _n n_3}&{}-\sqrt{\alpha _n n_3}&{}\sqrt{4\beta _n n_4}\\ 0&{}0&{}0&{}0 &{}0&{}0&{}\sqrt{\alpha _n n_3}&{}-\sqrt{4\beta _n n_4} \end{array} \right] , \end{aligned}$$

and

$$\begin{aligned} S^{(1:10)}_\text {Na}=&\frac{1}{\sqrt{M_\text {ref}}}\left[ \begin{array}{cccccccccc} -\sqrt{\alpha _h m_{00}}&{} \sqrt{\beta _h m_{01}}&{}-\sqrt{3\alpha _m m_{00}}&{}\sqrt{\beta _m m_{10}}&{} 0&{}0&{}0&{}0&{}0&{}0\\ 0&{} 0&{}\sqrt{3\alpha _m m_{00}}&{}-\sqrt{\beta _m m_{10}}&{} -\sqrt{\alpha _h m_{10}} &{} \sqrt{\beta _h m_{11}} &{}-\sqrt{2\alpha _m m_{10}}&{}\sqrt{2\beta _m m_{20}}&{}0&{}0 \\ 0 &{}0 &{}0&{}0&{}0&{}0&{} \sqrt{2\alpha _m m_{10}} &{}-\sqrt{2\beta _m m_{20}}&{}-\sqrt{\alpha _h m_{20}}&{}\sqrt{\beta _h m_{21}}\\ 0 &{}0&{}0&{}0&{}0&{} 0&{}0&{}0 &{}0&{}0\\ \sqrt{\alpha _h m_{00}}&{}-\sqrt{\beta _h m_{01}}&{}0&{}0&{}0&{} 0&{}0&{}0&{}0&{}0 \\ 0&{}0&{}0&{}0&{}\sqrt{\alpha _h m_{10}}&{}-\sqrt{\beta _h m_{11}}&{}0&{}0&{}0&{}0 \\ 0&{}0&{}0&{}0&{}0 &{}0&{}0&{}0&{}\sqrt{\alpha _h m_{20}}&{}-\sqrt{\beta _h m_{21}} \\ 0&{}0&{}0&{}0&{}0&{} 0&{}0&{}0&{}0&{}0 \\ \end{array} \right] \\ S^{(11:20)}_\text {Na}=&\frac{1}{\sqrt{M_\text {ref}}}\left[ \begin{array}{cccccccccc} 0&{}0&{}0&{}0&{}0&{}0&{}0&{}0&{}0&{}0\\ 0&{}0&{}0&{}0&{}0&{}0&{}0&{}0&{}0&{}0\\ -\sqrt{\alpha _m m_{20}}&{} \sqrt{3\beta _m m_{30}}&{}0&{}0 &{}0&{}0&{}0&{}0&{}0&{}0\\ \sqrt{\alpha _m m_{20}}&{} -\sqrt{3\beta _m m_{30}} &{}-\sqrt{\alpha _h m_{30}} &{}\sqrt{\beta _h m_{31}}&{}0&{}0&{}0&{}0&{}0&{}0\\ 0&{}0&{}0&{} 0&{}-\sqrt{3\alpha _m m_{01}} &{}\sqrt{\beta _m m_{11}}&{}0&{}0&{}0&{}0\\ 0&{}0&{}0&{}0&{}\sqrt{3\alpha _m m_{01}}&{} -\sqrt{\beta _m m_{11}}&{}-\sqrt{2\alpha _m m_{11}}&{}\sqrt{2\beta _m m_{21}}&{}0&{}0\\ 0&{}0&{}0&{}0 &{}0&{} 0&{}\sqrt{2\alpha _m m_{11}}&{}-\sqrt{2\beta _m m_{21}}&{}-\sqrt{\alpha _m m_{21}}&{}\sqrt{3\beta _m m_{31}}\\ 0&{}0&{}\sqrt{\alpha _h m_{30}}&{}-\sqrt{\beta _h m_{31}} &{}0&{}0&{}0&{}0&{}\sqrt{\alpha _m m_{21}}&{}-\sqrt{3\beta _m m_{31}}\\ \end{array} \right] , \end{aligned}$$

where $S^{(i:j)}_\text {Na}$ is the i$^{th}$-j$^{th}$ column of $S_\text {Na}$.

Note that each of the 8 columns of $S_\text {K}$ corresponds to the flux vector along a single directed edge in the $\hbox {K}^{+}$ channel transition graph. Similarly, each of the 20 columns of $S_\text {Na}$ corresponds to the flux vector along a directed edge in the $\hbox {Na}^{+}$ graph (cf. Fig. 10). Factors $M_{\text {ref}}=6000$ and $N_{\text {ref}}=1800$ represent the reference number of $\hbox {K}^{+}$ and $\hbox {Na}^{+}$ channels from Goldwyn and Shea-Brown’s model (Goldwyn and Shea-Brown 2011).

### Numerical steps to calculate the interphase intervals

For the 14-dimensional HH system we extract the deterministic phase as follows.

- Given a sample trajectory ${\mathbf {X}}(t)$, we approximate the deterministic infinitesimal phase response curve (iPRC) near the limit cycle, ${\tilde{{\mathbf {Z}}}}({\mathbf {X}}(t))$, by using the phase response curve on the deterministic limit cycle
  31 $$\begin{aligned} {\tilde{{\mathbf {Z}}}}({\mathbf {X}}(t))\approx {\hat{{\mathbf {Z}}}}({\mathbf {X}}(t)){\mathop {=}\limits ^{\varDelta }}{\mathbf {Z}}\left( \underset{s}{{\text {argmin}}} \left| \bigg (\gamma (s)-{\mathbf {X}}(t)\bigg )^\intercal {\mathbf {Z}}(s)\right| \right) ,\nonumber \\ \end{aligned}$$
  where $\gamma$ is a point on the deterministic limit cycle and ${\mathbf {Z}}$ is the infinitesimal phase response curve on the limit cycle (LC).
- To calculate the interphase interval, we first set up an isophase section (${\mathcal {S}}_0$) that intersects the deterministic limit cycle at the point ($\gamma _0$), such that for all ${\mathbf {X}}(t) \in {\mathcal {S}}_0$, ${\hat{{\mathbf {Z}}}}({\mathbf {X}}(t))={\mathbf {Z}}(\gamma _0)$. The point $\gamma _0$ is chosen to coincide with the upcrossing of the Schmitt trigger voltage $V_{th}$ by the deterministic limit cycle. Given a specific initial condition, there is a one to one correspondence between points on the deterministic limit cycle, the time before it reaches the end of the limit cycle, and the phase response curve. We select the isophase section as a set of points that share the same iPRC value, which guarantees the same return time for all points on the same isophase section.
- Similarly as the one-dimensional case, the times that a trajectory crosses a given isophase section are recorded, and the time between consecutive crossing times are recorded as the interphase intervals (IPIs). Numerically to do so, we assign an index for each point on the LC, starting from 1 at the initial point. When we use Eq. (31) to estimate the iPRC, we also return the index of the point on LC. Note that the isophase section (${\mathcal {S}}_0$) can be identified either by the phase response curve (${\mathbf {Z}}(\gamma _0)$) on the LC, or its corresponding index on the LC. Here, we are collecting the indices of iPRC (i.e., index of *s* in Eq. (31) on the LC) for each point on the sample trajectory. In each full oscillation, we mark the first time that the indices recorded for the trajectory cross a specific index (this is the threshold) as the time for the IPIs, and linear interpretation is used to approximate the exact threshold-crossing time.

## Footnotes

1 The convergence of the MRT isochron function to the deterministic asymptotic phase function in the limit of vanishing noise was not addressed in Cao et al. (2020), and a rigorous investigation of this question lies beyond the scope of the present paper. In §3.2-3.3, we will simply assume that the MRT isochrons converge to the deterministic isochrons, so that we can approximate one via the other in specific cases.

2 For a Markov process as in (2), if it is started in the far past at a long-forgotten initial condition, we can assume that passage times between the curves $\ell _i$ follow a stationary statistics.
